# TIBA: A web application for the visual analysis of temporal occurrences, interactions, and transitions of animal behavior

**DOI:** 10.1371/journal.pcbi.1012425

**Published:** 2024-10-25

**Authors:** Nicolai Kraus, Michael Aichem, Karsten Klein, Etienne Lein, Alex Jordan, Falk Schreiber

**Affiliations:** 1 Department of Computer and Information Science, University of Konstanz, Konstanz, Germany; 2 Behavioural Evolution Research Group, Max Planck Institute of Animal Behavior, Konstanz, Germany; 3 Faculty of Information Technology, Monash University, Melbourne, Australia; Institut des sciences cognitives Marc Jeannerod, FRANCE

## Abstract

Data in behavioral research is often quantified with event-logging software, generating large data sets containing detailed information about subjects, recipients, and the duration of behaviors. Exploring and analyzing such large data sets can be challenging without tools to visualize behavioral interactions between individuals or transitions between behavioral states, yet software that can adequately visualize complex behavioral data sets is rare. TIBA (The Interactive Behavior Analyzer) is a web application for behavioral data visualization, which provides a series of interactive visualizations, including the temporal occurrences of behavioral events, the number and direction of interactions between individuals, the behavioral transitions and their respective transitional frequencies, as well as the visual and algorithmic comparison of the latter across data sets. It can therefore be applied to visualize behavior across individuals, species, or contexts. Several filtering options (selection of behaviors and individuals) together with options to set node and edge properties (in the network drawings) allow for interactive customization of the output drawings, which can also be downloaded afterwards. TIBA accepts data outputs from popular logging software and is implemented in Python and JavaScript, with all current browsers supported. The web application and usage instructions are available at tiba.inf.uni-konstanz.de. The source code is publicly available on GitHub: github.com/LSI-UniKonstanz/tiba.

## Introduction

With the ever-increasing need to understand temporal aspects of behavior, the research field of animal behavior is currently in a transitional phase in terms of the type and amount of data collected [1–3]. Animal behavior is often video recorded and later quantified with free software such as CowLog [4] or BORIS [5]. These tools facilitate the logging of behavior and produce data frames where each row contains the time, the subject, optionally the recipient, and a description of the observed behavior. Existing tools for the automated annotation and classification of behavior include SimBA (Simple Behavioral Analysis [6]), MARS (The Mouse Action Recognition System [7]) and JAABA (Janelia Automatic Animal Behavior Annotator [8]).

One common approach to visualizing and analyzing animal behavior data is to analyze the amount, duration, or behavioral timeline of specific behaviors. It is also possible to visualize the temporal development of behavior, e. g. by accumulating counts separately for specific behaviors (or broader-scale behavioral categories), or for each individual over time. This type of analysis facilitates inspection of the expression of different behaviors during a given time period of observation, e. g. in the context of (dyadic) animal contests [9]; or examining co-occurrences of specific behaviors across a large number of individuals, e. g. during social (a)synchronization events [10]. A further approach to visualizing and analyzing social interaction data of animal behavior is via social networks (their construction and interpretation are extensively studied in [11–13], and the importance, methods and applications of network visualizations are well established in [14–16]), allowing the identification of differences between network members and/or relationships, e. g. in the context of studying group hierarchies [17]. The network approach also allows the study of behavioral sequences by visualizing behavioral transitions and their respective frequencies. However, there is very little software that allows the user to visualize, explore, and analyze these kinds of data together.

Our application TIBA targets behavior visualization by providing six different components: (1) a distinct behavior chart, (2) a behavioral timeline, (3) a temporal occurrences chart, (4) an interaction network and (5) a behavior transition network describing behavioral sequences, and (6) the comparison of the behavioral sequences by means of clustering pairwise distances. TIBA thus brings together different approaches to the visualizations of behavioral data, making them available to everyone regardless of platform and device, as TIBA may be both used from the running web instance or locally self-hosted.

In the following sections, we first introduce the sample data and look at the individual visualization methods in the design and implementation section, detailing their implementations and their application to the sample data. In the discussion, we deal with the biological significance of the individual visualization components and thereby discuss how TIBA may support common research questions via visualization methods. Further, we discuss the utility of TIBA compared to existing applications in the field of animal behavior analysis, showcasing the utility of our tool within this context. In the concluding section, future directions of TIBA, and visualization of animal behavior in general, are outlined.

## Design and implementation

**Fig 1:**
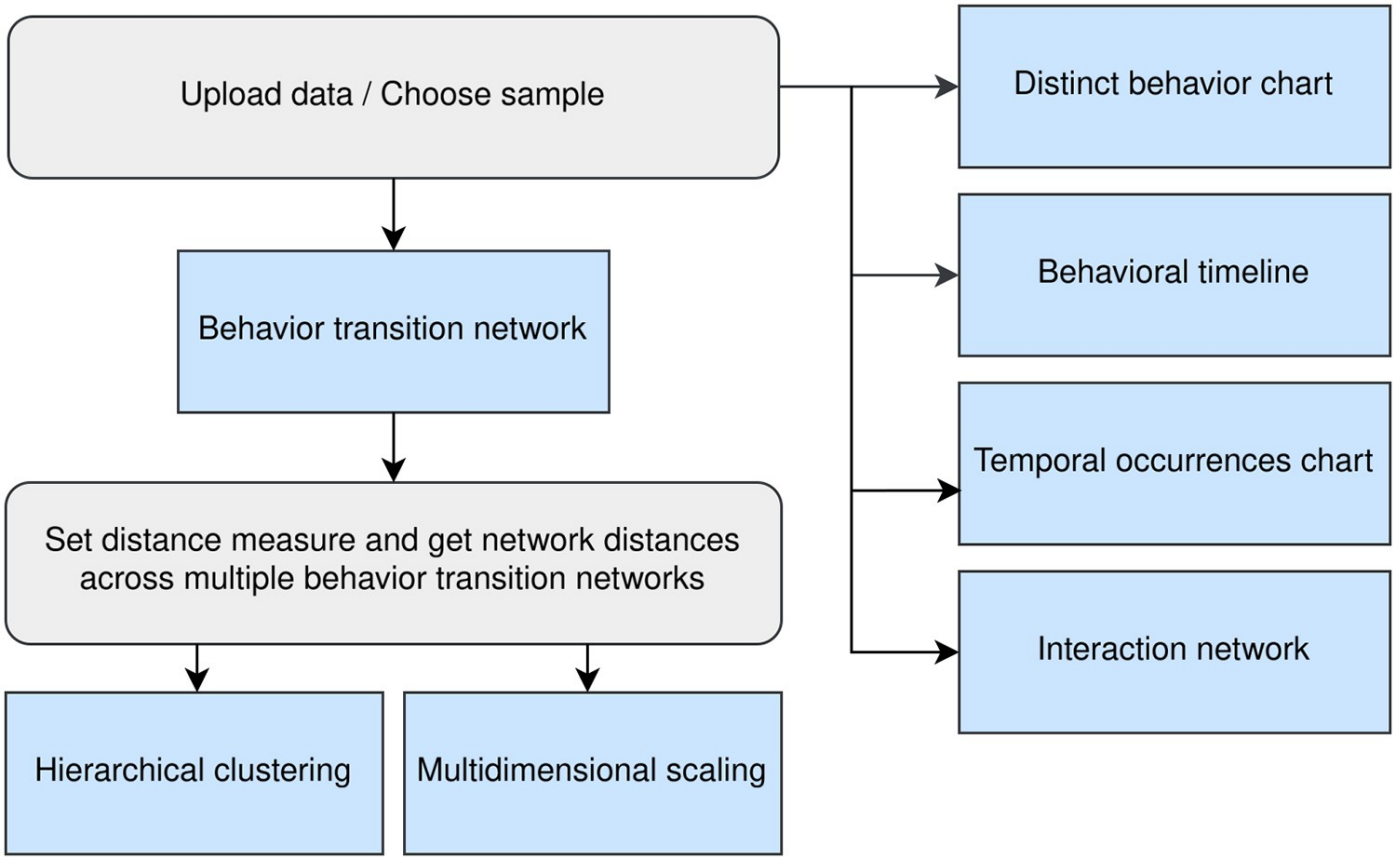
Workflow visualization of the application, illustrating the required steps and exportable outputs. Steps include data upload or sample selection, and distance measure selection. Blue nodes represent available visualizations, each may be further configured and exported.

When accessing the website, users are given the option to upload their own data or select one of three samples to test the functionality. Output from the event-logging software BORIS is natively supported, but any data in CSV or XLSX format that contains the columns *Time*, *Subject*, *Behavior*, *Status*, and optionally *Modifier* can be processed. An appropriate error message will be displayed if problems occur during data upload. On data upload or sample selection, general information like the name and unique values in the data are displayed and five interactive visualizations are generated and displayed. See the workflow in Fig 1.

For all visualizations, single individuals or behaviors (i. e. unique values from the columns *Subject* or *Behavior*) may be deselected and thus excluded from the computation. The interaction and behavior transition networks also have specific options for further customization. Changes are applied by pressing the respective buttons which trigger a new computation and visualization. For each of the visualizations, different download options are available. Furthermore, TIBA allows researchers to compare and cluster the behavior transition networks. In the following, we will give a detailed description of the available visualizations and the functionality to compare behavior transition networks.

### Sample data

The sample data were generated by one of the authors using BORIS and contains the logged behavior of 20 min video recordings from three cichlid species: *Lamprologus ocellatus, Neolamprologus multifasciatus and Telmatochromis temporalis*. These species are small fish of the tribe Lamprologini, native to Lake Tanganyika (Africa) that inhabit empty snail shells on the lake floor with conspecifics located at species-typical distances to each other. Due to this circumstance, they are commonly thought to exist on a broad spectrum, ranging from rather solitary to socially-living species. Given anecdotal field observations, which suggest that the three species exhibit a shared behavioral repertoire, this makes for a fascinating case to explore the behavioral mechanisms that gave rise to such divergent strategies of social living [18]. As a reference, the sample for *N. multifasciatus* can be downloaded from the website.

Data collection was performed using underwater cameras and caused minimal disturbance to the animals. This work adhered to the ASAB/ABS Guidelines for the Use of Animals in Research and field work was carried out with the permission of the Fisheries Department of Zambia under a study permits issued by the government of Zambia (SP008735 and SP2268511/9–21) and a memorandum of understanding (MOU 101/14/11), and adhered to the regulations of the Zambian Prevention of Cruelty to Animals act. These species are listed “Least Concern” on the IUCN Red List of Threatened Species.

### Distinct behavior chart

**Fig 2:**
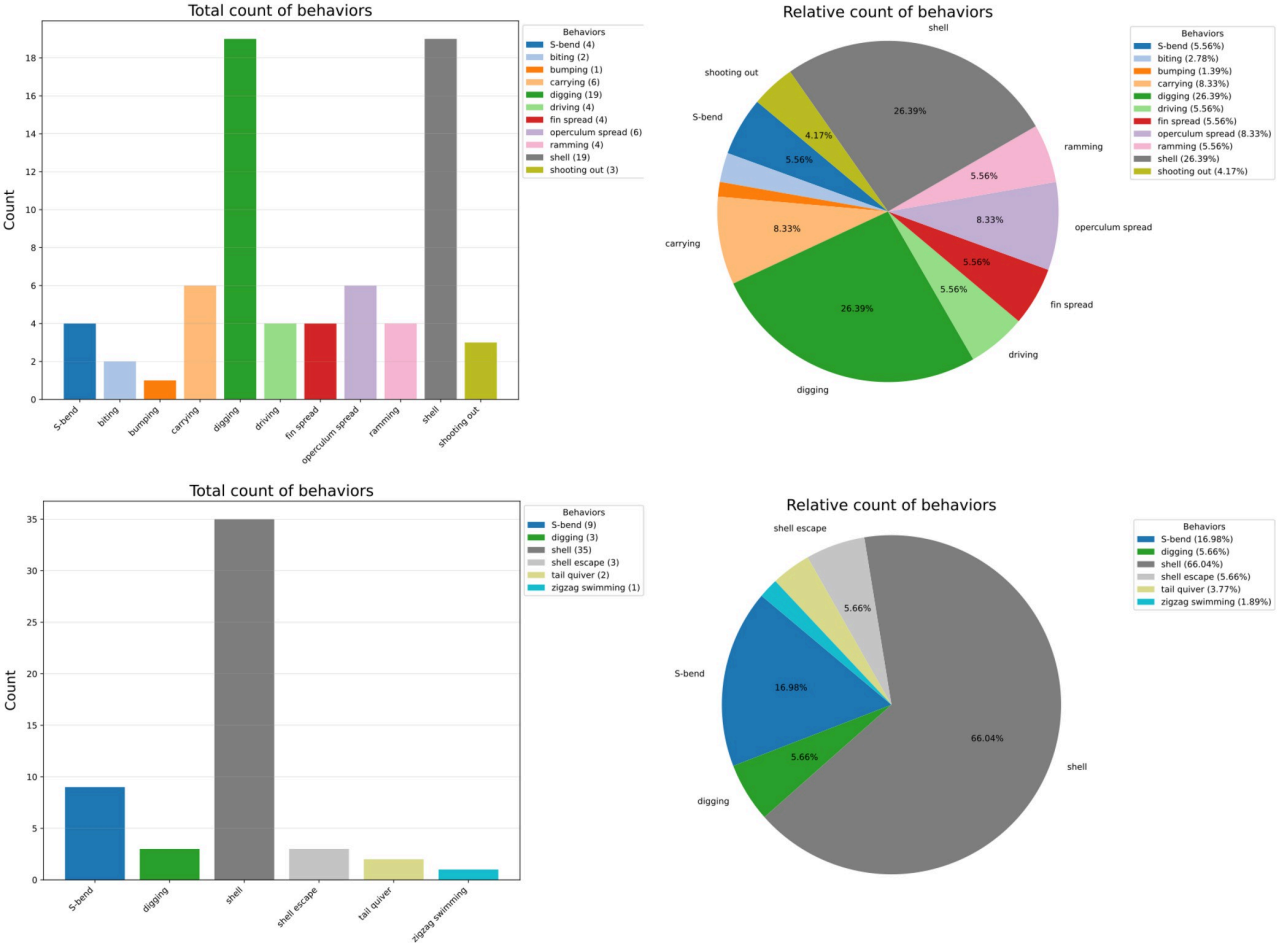
Distinct behavior charts for subject 1 (top left and right) and subject 5 (bottom left and right) from the sample data of *Neolamprologus multifasciatus* from the website (S1 Dataset). The total count for distinct behaviors is shown at the left, with the relative proportion per behavior on the right.

The distinct behavior chart visually represents the distribution of behaviors, offering options to display either the number of observations or their total duration as a bar chart. Alternatively, a relative display option presents the data as a pie chart. Behavioral categories can also be displayed instead of individual behaviors. Individuals, behaviors, or behavioral categories (if selected) can be excluded from the calculation by clicking the button representing the respective individual or behavior. The charts are constructed using the plotting library matplotlib [19], see also Fig 2.

### Behavioral timeline

**Fig 3:**
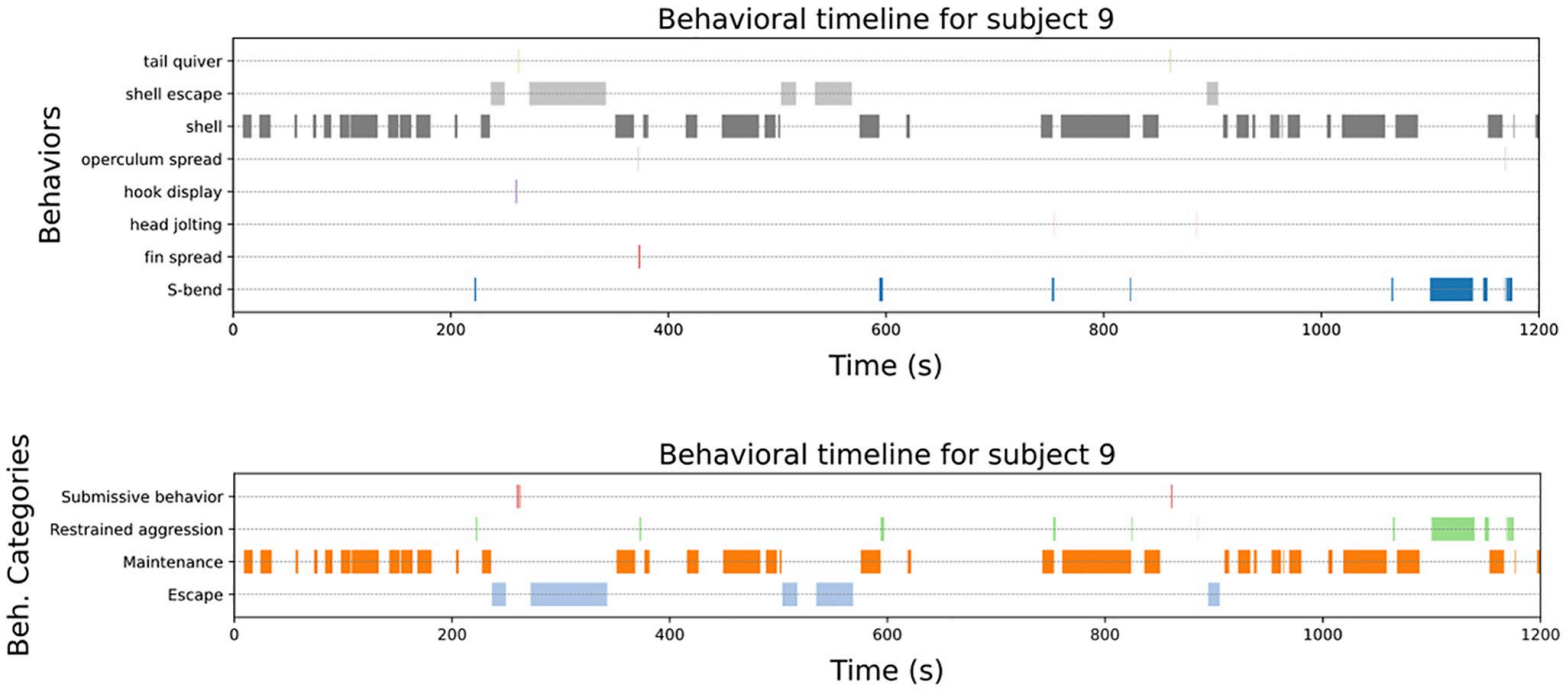
Behavioral timelines for subject 9 from the sample data of *Neolamprologus multifasciatus* from the website (S1 Dataset). The timeline for the behaviors is shown above. They describe the same data as Fig 2. The timeline for the corresponding behavioral categories is directly below.

The behavioral timeline shows the time and duration of observed behavior. For this purpose, the time is shown on the x-axis and each individual behavior is shown as a line, with a colored horizontal bar indicating when a behavior occurs. Behavioral categories can also be displayed instead of individual behaviors. Individuals, behaviors, or behavioral categories (if selected) can be excluded from the calculation by clicking the button representing the respective individual or behavior. The charts are constructed using the plotting library matplotlib [19], see also Fig 3.

### Temporal occurrences chart

**Fig 4:**
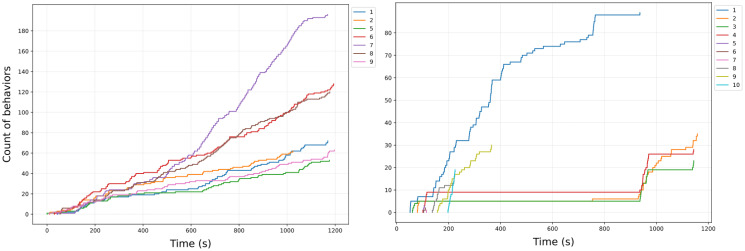
Temporal occurrences charts generated from sample data from the website showing the time on the x axis and the cumulative count of selected behaviors per individual on the y axis. Data for the two shown plots originate from two different recordings: *Neolamprologus multifasciatus* (S1 Dataset) on the left and *Lamprologus ocellatus* (S2 Dataset) on the right side, each documenting behavior over a 20-minute period. There are 7 individuals recorded on the left and 10 on the right.

The temporal occurrence chart combines the two previous charts in the sense that it shows the temporal accumulation of behavior. For this purpose, the time is shown on the x-axis and the cumulative count of selected behaviors shown up to that time (separately for each individual) on the y axis. Alternatively, one line per behavior per individual can be shown. Behavioral categories can also be displayed instead of individual behaviors. Individuals, behaviors, or behavioral categories (if selected) can be excluded from the calculation by clicking the button representing the respective individual or behavior. The charts are constructed using the plotting library matplotlib [19], see Fig 4. An interpretation of the charts follows in the results and discussion section.

### Interaction network

**Fig 5:**
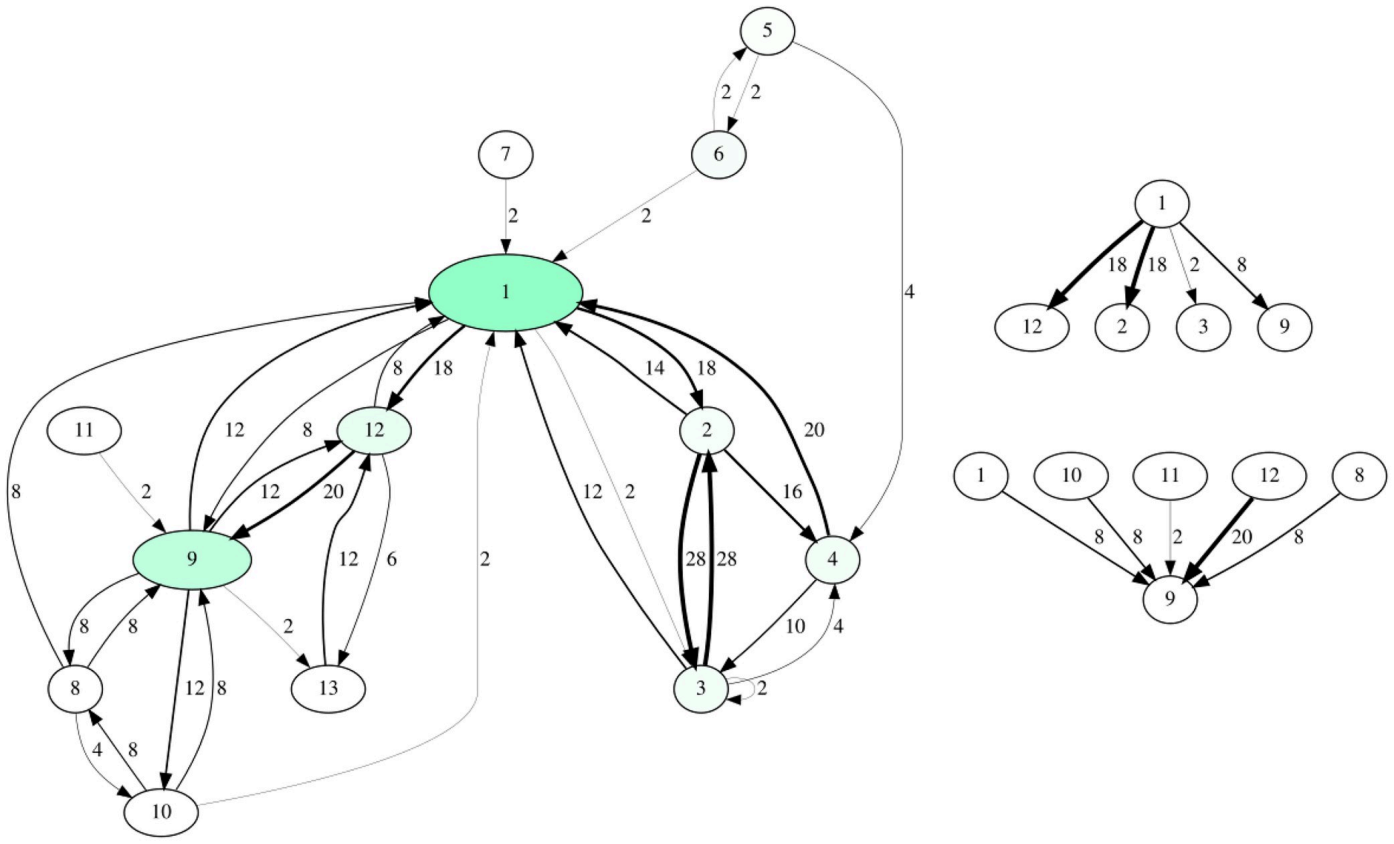
Interaction networks generated from the sample data of *Lamprologus ocellatus* from the website (S2 Dataset). On the left side, all interactions i. e. behavior emanating from one individual and directed towards another are displayed, the betweenness of nodes (individuals) is mapped to node size and color brightness. On the right side detailed views for behavior emanating from subject 1 (top) and targeting subject 9 (bottom) are depicted.

The interaction network displays the number and direction of interactions between individuals (see Fig 5). It is a directed weighted network where nodes represent individuals and edges are drawn from individual A to individual B if A is the subject of a behavior and B is the recipient (i. e. the corresponding value in the column *Modifier*). The number of interactions determines the weight of an edge, which is displayed as edge label and mapped to the edge width. Individuals emanating and receiving behavior may be deselected separately and a weight threshold for edges to be displayed may be set. Node attributes and centrality metrics are calculated for the network, which describe the number of incoming and outgoing edges (interactions with different individuals) for each node (individual), as well as the sum of their labels (number of interactions) and four centralities (in-degree, out-degree, closeness and betweenness centrality). Degree centrality quantifies, by summing up the number of edges, the direct connections (i.e., relationships) a focal node (= focal individual) has with other nodes (= members of the social network). The number of connections is considered indicative of an individual’s influence on group members and possibly the whole network [11, 20]. In-degree refers to the number of incoming edges and reflects from how many group members the individual receives behaviors; whereas out-degree refers to the number of outgoing edges and reflects to how many group members the individual is exhibiting behaviors. Closeness centrality quantifies the reciprocal of the average distance from a focal node (= focal individual) to all other nodes (= group members) in the network [21]. This metric thus takes into account both direct as well as indirect connections (= relationships) and is thought to better reflect the potential influence the individual has on the entire group [20]. Betweenness centrality quantifies the fraction of shortest paths (i.e., fewest possible number of consecutive edges) between pairs of nodes in the network which include the focal node (= focal individual) [22]. Betweenness therefore reflects the importance of the focal individual as a point of social connection and transfer between different sections of the network [11, 20]. Furthermore, individuals with high betweenness centrality are thought to be important for group stability as their removal may lead to a fragmentation of the network into smaller subunits [23–25].

The four centralities can be visually represented through a mapping to node size and color brightness, allowing users to select a color for the latter option. The node attributes and centralities can be exported and also be sorted by clicking on the respective attribute or centrality. The interaction networks are computed with networkx [26], layout and visualization of the respective graphs is achieved with GraphViz [27].

### Behavior transition network

**Fig 6:**
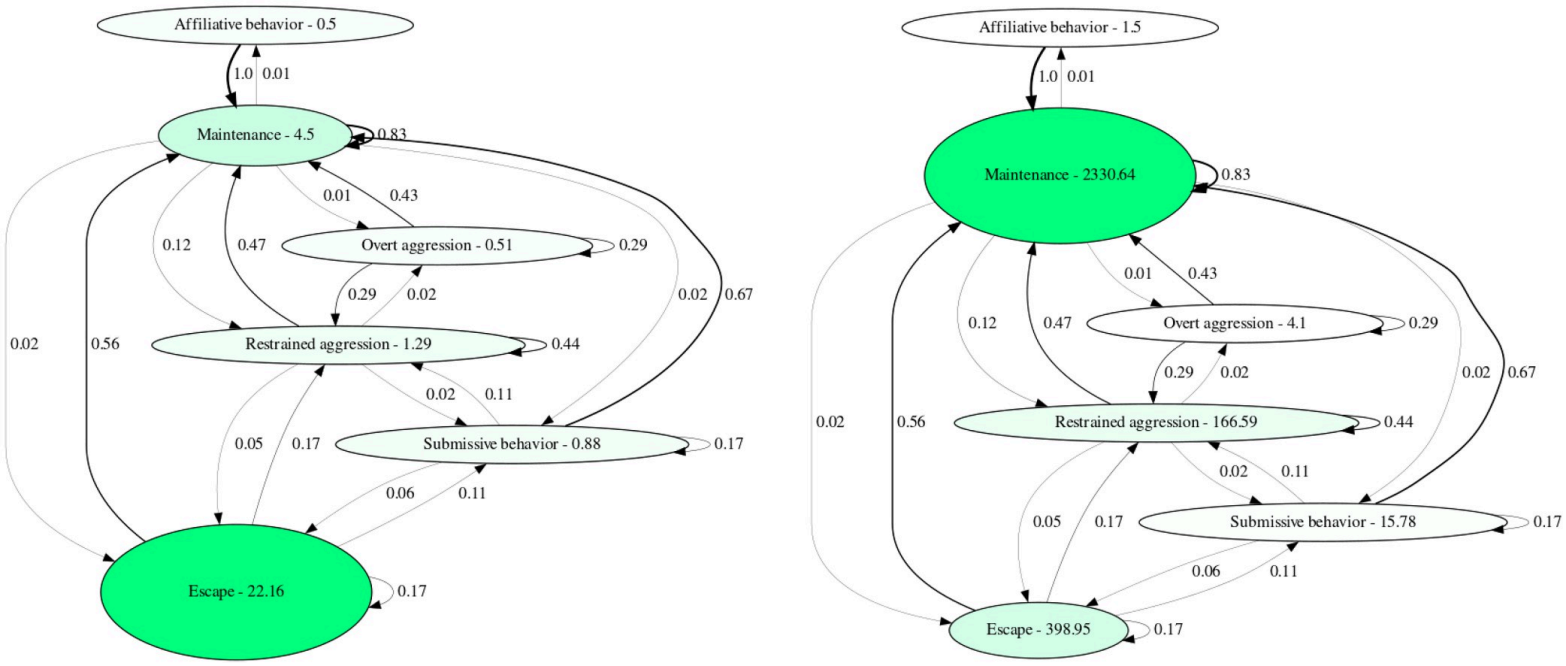
Behavior transition networks generated from the sample data of *Neolamprologus multifasciatus* from the website (S1 Dataset). Both are set to use behavioral categories as nodes. On the left hand side, the average time a behavioral category is shown is mapped to node label, size and color brightness, while on the right side the total time is applied as mapping. The node properties are set as linear normalized. Edge values depict the transitional frequency from one category to another, rounded to the second digit. The edge width is set to be dependent on the edge weighting.

The behavior transition network displays temporal sequences of behavioral events. It is visualized by a directed, weighted network where the nodes represent either behavioral events or behavioral categories and edges represent the transition from one behavior to another (see Fig 6). That is, an edge describes a behavioral transition or sequence (consecutive display of behavior). A self-loop occurs if a behavior is displayed, halted and then displayed again without any intermediate other behavior.

The behavior transition network is generated as follows. First, behavior is considered separately for all individuals (unique values in column *Subject*), allowing the temporal sequence of behavior per individual to be read from successive rows. From this, a list consisting of 3-tuples (ordered lists of three elements) is generated: the first two elements being observed behaviors and the third showing the count of behavioral successions i. e. how often the second behavior was shown by the individual directly after the first behavior. These lists are accumulated for all selected individuals to derive the edge weights and are then visualized as the behavior transition network. A threshold for the edge weights may be set such that edges with weights below are excluded from the visualization (but still included in the calculation to avoid distorting the transitional frequencies). Individual behaviors, behavioral categories or individuals may be deselected and thereby excluded from the calculation and visualization. Following further customizations are available:

**Node values** Set either unique behaviors or behavioral categories (if present) as node values.
**Edge values** Set either the absolute number of transitions or the relative transitional frequencies (i. e. the relative frequency with which a certain behavior follows another behavior) as edge weights. In the latter case, for each unique edge, the ratio of its weight and the sum of the weights of all other outgoing edges from the same node is taken to derive the frequency of the respective behavioral sequence.
**Color setting** Select one of two coloring options: either one color is set and the nodes differ in the color saturation depending on the chosen mapping or each node and its outgoing edges are mapped to colors according to their behavioral category. In the latter case, the options for color hue and color saturation are disabled.
**Edge width** Select whether the width of drawn edges is fixed or dependent on the weights. In the latter case one may choose an edge width factor to control the edge width range.
**Normalization** Select whether edge width, node size, and node saturation are normalized in either a linear or logarithmic fashion.
**Node size/color saturation/label mapping** Node appearance may be altered by mapping the total count, the average time or the total time or any of the calculated centralities of a behavioral event to the node size, the node color saturation or onto a label inside the node.

Node attributes, which describe for each node (behavior) the total and average time it is observed, the amount of occurrences, the amount of ingoing and outgoing edges (distinctive behavioral transitions), as well as four centralities (in-degree, out-degree, closeness and betweenness centralities [28]) are calculated and can be exported, sorted and mapped in the same way as in the interaction networ. The in-degree centrality quantifies the number of incoming edges (transitions) to a node (behavior). Behaviors with high in-degree centrality have a variety of possible preceding behaviors. The out-degree centrality quantifies the number of outgoing edges (transitions) from a particular node (behavior). Behaviors with high out-degree centrality have a variety of possible subsequent behaviors. The closeness centrality considers the average distance from a node (behavior) to all other nodes (behaviors). Closeness centrality in a behavior transition network assesses how quickly a behavior can be reached from other behaviors in terms of behavioral transitions. Betweenness centrality measures the frequency with which a node (behavior) lies on the shortest paths between pairs of other nodes (behaviors). Betweenness centrality in a behavior transition network identifies behaviors that act as critical connectors between other behaviors.

The behavior transition networks are computed with networkx [26], layout and visualization of the respective graphs is achieved with GraphViz [27].

### Comparing behavior transition networks

**Fig 7:**
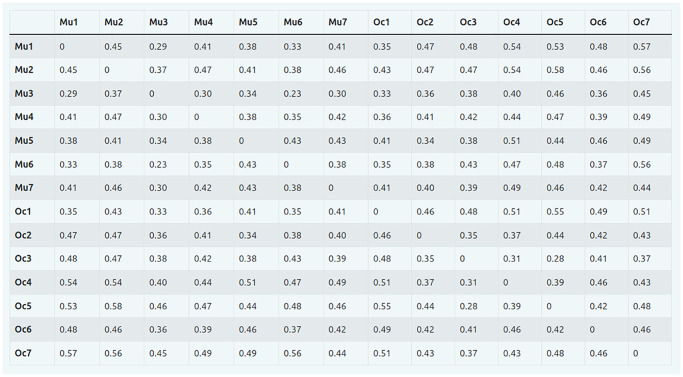
Distance matrix from pairwise comparisons of behavior sequences of the two cichlid species *Neolamprologus multifasciatus* and *Lamprologus ocellatus* (S4–S17 Datasets), using the Network Portrait Divergence as distance measure. As distances are symmetric and a network has a distance of zero to itself, the distance matrix is itself symmetric with zeros on the diagonal.

**Fig 8:**
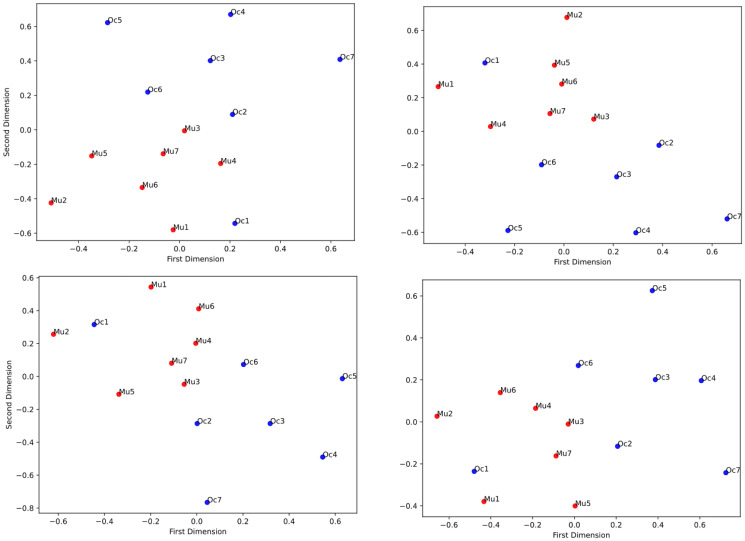
Two-dimensional embeddings of distances between transition networks as result of multi-dimensional scaling (MDS) applied to the distance matrix (Fig 7), where distances between single data points are preserved as good as possible while reducing dimensions. Data of two cichlid species, *Neolamprologus multifasciatus* and *Lamprologus ocellatus* (S4–S17 Datasets), are compared pairwise with the Network Portrait Divergence applied as distance measure. The indices correspond to the first letters of the species, where the appended number allows the assignment to the same record in the dendrogram in Fig 9. The four embeddings were created with the parameter n_init set to 400 (the number of times the algorithm runs) and with the starting states 0, 40, 65 and 77 from the top left to the bottom right corner.

**Fig 9:**
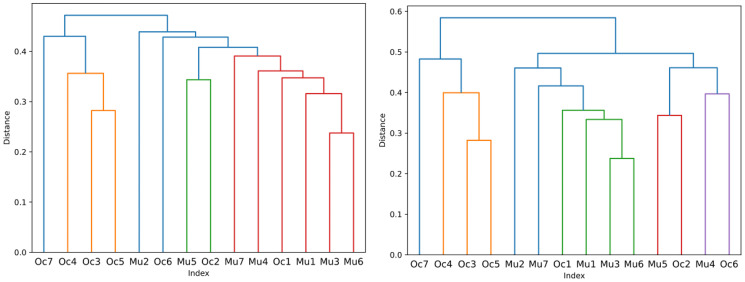
Dendrograms of distances across transition networks as a result of agglomerative clustering of the distance matrix (Fig 7). The average and complete distances between clusters are set as linkage criteria, on the left and on the right, respectively. Merged clusters with distances below 0.4 are set to have distinct colors. Data of two cichlid species, *Neolamprologus multifasciatus* and *Lamprologus ocellatus* (S4–S17 Datasets), are compared pairwise and the Network Portrait Divergence is applied as distance measure. The indices correspond to the first letters of the species, where the appended number allows the assignment to the same record in the plot in Fig 8.

To facilitate the analysis of behavioral relationships between different species (or individuals), TIBA also provides methods to compare and cluster different behavior transition networks. Multiple data sets can be uploaded, for each of which a separate behavior transition network is generated. Pairwise comparison of these networks can now be done by one of the available distance measures, which effectively is an algorithm that computes a continuous similarity score for two provided networks. After selecting a distance measure, networks are compared pairwise. The resulting distance matrix (see Fig 7) can be exported or users can apply multidimensional scaling (to embed the distances into two dimensions, see Fig 8), or hierarchical clustering (see Fig 9). The following functionalities are available:

**Choose network distance measure** Available measures are the Network Portrait Divergence [29], the Frobenius norm, the Hamming weight, the Distributional Non-backtracking Spectral Distance [30], the Ipsen-Mikhailov Distance [31] and the Degree Divergence (see also [32]). More information on their exact definition and computation can also be found in the documentation of networkx [26].
**Output** Three outputs (indexed corresponding to the upload indexing) are generated: a distance matrix, a 2D embedding using multidimensional scaling and a hierarchical clustering tree. The first two characters of the filenames are used for indexing, optionally enumerated.

Multidimensional Scaling (MDS) is a statistical technique used to visualize the relative similarities or dissimilarities among a set of objects based on a distance matrix. First, the distance matrix is computed with the distance measure selected by the user. MDS then reduces the dimensionality of the data to a lower-dimensional space (two dimensions), thereby aiming to preserve the original distances between elements (in our case networks) as much as possible. The reduced-dimensional representation obtained through MDS is then plotted in a scatterplot, where each point represents a behavior transition network. The distances between points in the plot reflect the dissimilarities between the corresponding networks in the original data. Networks that appear close together in the MDS plot have similar transition patterns between behaviors. Networks that are distant from others may represent unique or distinct behavioral states or patterns. The arrangement of points in the plot may suggest transitional pathways between different behavioral states or clusters. Two parameters are available: A starting state which is necessary to initialize the algorithm. The same starting state guarantees the exact same output across different runs but one must test different starting states and verify that the emerging patterns in the output stay consistent. Only a pattern that emerges consistently across different starting states should be considered. Also, the number of times the algorithm runs (n_init) may be set to find a good balance between the running time and the precision of the results.

Hierarchical (agglomerative) clustering is a method used to cluster similar objects into groups based on their pairwise similarities or dissimilarities. Similar to MDS, a distance matrix serves as the basis for the clustering. Hierarchical clustering proceeds by iteratively merging clusters until all objects belong to a single cluster. The applied agglomerative clustering algorithm uses linkage criteria to determine the distance between clusters. Three linkage criteria are selectable by the user: Average linkage, complete linkage and single linkage. Average linkage computes the average distance between all pairs of points in two clusters. It tends to produce clusters with more uniform sizes. Complete linkage measures the maximum distance between any pair of points in two clusters. It tends to create compact clusters, suitable for identifying distinct groups. Single linkage determines the distance between the closest points (minimum distance) of two clusters. It tends to form clusters with elongated or chain-like shapes. The output may also be parameterized by setting a distance threshold for different coloring of the branches to indicate close distances. The resulting dendrogram or cluster tree shows the hierarchical relationships between clusters. Distinct clusters or branches in the dendrogram may correspond to species or groups of individuals exhibiting specific behavioral characteristics. Further, the branching structure of the dendrogram can reveal hierarchical relationships between different behavioral clusters, indicating transitions between different levels of behavioral complexity.

In summary, multidimensional scaling and hierarchical clustering offer powerful tools for exploring the structure and relationships within behavior transition networks, allowing researchers to uncover patterns and associations that may not be apparent through other analytical approaches. Both visualizations are computed using scikit-learn (sklearn) [33] and visualized with matplotlib [19].

### Exporting images and networks

All visualizations may be exported from the web application as Scalable Vector Graphics (SVG). The interaction and behavior transition networks are also available as Grapviz Dot (GV) [34] and Graph Modelling Language (GML) [35] files. The node attributes and centralities for the interaction and behavior transition networks may be exported as Comma-Separated Values (CSV) files.

### Implementation

TIBA is hosted as a web application at the University of Konstanz and works on all current browsers. It is implemented using open source software. The web server is implemented using Python 3.8+ and django [36], using matplotlib [19] for plotting and networkx [26] for network computation while the layout and visualization of the respective graphs is achieved with GraphViz [27]. The User Interface is written in ReactJS [37]. The user data is sent to the API with each request which is answered with the respective image and network files to display and download.

## Results and discussion

We introduced a web application that enables researchers to visually explore six different aspects of animal behavior based on the provided data. These are (1) distinct behaviors, (2) behavioral timelines, (3) temporal occurrences, (4) interactions, (5) behavioral transitions (behavioral sequences), and (6) the comparison of the latter. In this section, we will discuss these components with regard to specific research questions from the area of animal behavior.

The first two components allow an initial exploration of the overall pattern of behaviors, both as raw behavioral counts and as percentage of the overall behavior exhibited during the period of observation. These approaches are long-standing in animal behavioral studies and are a common approach to comparing basic data categories. For example, Fig 2 shows that individuals 1 and 5 have different behavioral repertoires, both in terms of the number of different expressed behaviors (11 vs. 6) as well as with regard to the broad categories of expressed behaviors: while individual 5 in the context of aggression appears to exclusively resort to restrained aggressive behaviors (“S-Bend”), individual 1 additionally exhibits overt aggressive behaviors (“ramming”). Furthermore, individual 5 exhibits shell escapes, a behavior occurring in response to con- or heterospecific threats, whereas individual 1 does not. Taken together, these broad behavioral differences between the two individuals may reflect the distinct environmental and social pressures associated with occupying different ranks in hierarchical social groups.

The third component of our tool, the temporal occurrences chart, plots the cumulative count of selected behaviors per individual and therefore enables researchers to explore the long and medium term temporal dynamics of behaviors and interactions among multiple individuals, an essential component of many aspects of animal behavior, e. g. in the context of contest dynamics, where temporal aspects of behavior allow researchers to determine when contests are in escalation or de-escalation phases [9]. The visual exploration of behavioral synchronization also facilitates insight into behavioral dependency over shorter timescales. For example, Fig 4, right shows a striking synchronization among individuals 2, 3 and 4, whereas individual 1 acts more independently. In the context of predator escaping behavior, this pattern could for example indicate within-group differences in information transmission, divergent risk strategies, or a partitioning of labor among members of the social group—all of which could be tested in subsequent experiments.

The fourth component, the generation of interaction network visualizations, facilitates e. g. a first identification of central individuals within a social group. For instance, in our application example (Fig 5), the network intuitively shows individuals 7 and 11 having lower degree centrality (fewer incoming and outgoing interactions) as compared to all other members of the interaction network, which may lead to further inquiries into the relationship between social and physical space occupied by each group member.

The fifth component, the visualization of behavioral transitions, allows ethologists to identify behavioral biases or stereotyped behavior within individuals, examine common notable behavioral motifs over time or among individuals, and to inspect whether or not there is a consistent succession of behaviors [9]. In our application example (Fig 6), an inverse relationship between average and total time allocated to escaping vs maintenance behavior is apparent, which may indicate risk-sensitive adjustments in time budgets that depend on the interaction of biotic and abiotic environmental factors perceived by the group.

The sixth component, the comparison and clustering of the behavior transition networks, facilitates the analysis of behavioral phenotypes on a broader scale, i. e. between different species or groups. In our application example (Figs 7 and 9) there is an overall pattern that our two different species fall into separate clusters of behavioral transitions. However, one behavioral observation from *L. ocellatus* clusters with those of *N. multifasciatus* across all runs, irrespective of the chosen starting state (as depicted in Fig 8). This is also visible in the dendrograms in Fig 9, that yielded an overall resembling pattern, despite the different linkage criteria. These results demonstrate that under certain circumstances the behavioral motifs of different species can be more or less similar. Such an observation can provide a powerful starting point to generating hypotheses about how behavior might evolve or be expressed in different species, for example whether species flexibly combine common behavioral components into divergent behavioral motifs, instead of possessing different behavioral repertoires *per se*, a difference that could lead in turn to divergence in social organization [18].

Overall, the consolidation of these six main components into an interactive system provides a versatile and sophisticated way to explore differences not just in the behaviors animals express, but in the social and temporal aspects of this expression, facilitating exploration and hypothesis generation even when faced with increasingly complex behavioral data sets.

## Existing tool landscape

In this section, we discuss existing applications for the analysis and visualization of animal behavior and highlight unique features of our application TIBA. The established tool for human-annotated quantification of animal behavior is BORIS (Behavioral Observation Research Interactive Software [5]). SimBA (Simple Behavioral Analysis [6]) is an open source toolkit for computer classification of complex social behaviors in experimental animals. MARS (The Mouse Action Recognition System [7]) is a pipeline for pose estimation and classification of social behavior in videos of interacting mice. JAABA (Janelia Automatic Animal Behavior Annotator [8]) is a machine learning-based system that enables researchers to automatically compute quantitative statistics describing video of behaving animals. While these tools perform well in classifying behavioral data, their visualizations are based on two charts: a behavioral timeline that shows the time period in which behaviors are observed, and a chart depicting the count (or total time) of behaviors observed. However, different types of networks can also be obtained from behavioral data, such as an interaction network that indicates directed behavior between individuals or a behavior transition network that describes behavioral sequences and respective transitional frequencies (indicating the frequency of transitions from one behavior to another in a sequence of behaviors exhibited by individuals). The latter is for example tackled by Behatrix [38], a tool that enables the analysis and comparison of behavioral sequences. Regarding the behavioral sequence analysis, Behatrix and TIBA follow a similar idea, but they still differ in some fundamental aspects. While both tools allow for the customization of the resulting visualizations, Behatrix provides users with a programmatic interface to the underlying source code, while TIBA provides a graphical user interface that abstracts some of the visual attributes. The available distance measures for networks differ between the tools, as do the additional features: TIBA provides the visualization of parameters that compose behavior sequences (normalization of node and edge size, mapping of total and average time to node size, color and label, or setting a unique color for each node and respective outgoing edges). Generated transition networks may be further compared using different distance measures from the software package netrd [32] and the resulting distances can either be visualized in a novel way as 2D embedding using multidimensional scaling or clustered hierarchically using the respective functions from scikit-learn (sklearn) [33].

Through the user-friendly selection of individual behaviors and individuals, TIBA enables a fine-grained analysis that is not possible with other tools. It also brings new functionality into the landscape of related tools through the centrality analysis of nodes in the interaction and behavior transition networks and especially the comparison of the latter, enabling researchers to better understand social interactions and behavioral motifs. Further, natural groupings and delineations in the visualization of the distances across different behavior transition networks can contribute to the analysis of behavioral sequences, and behavior in general, of the individuals, groups, or species under investigation.

In summary, TIBA integrates behavioral data visualizations within a user-friendly web interface and facilitates the generation and exploration of interaction and behavior transition networks, offering versatile comparison and visualization methods.

## Availability and future directions

The web application and usage instructions are available at tiba.inf.uni-konstanz.de.

There are several directions in which TIBA could be extended. First, one might add graph distance measures for the comparison of behavior transition networks. An option would be to apply a Mantel-test [39] on the adjacency matrices of two networks to derive a distance. Furthermore, it would be interesting to investigate not only two consecutive behaviors, but the occurrences of longer chains of behaviors [40]. As the study of animal behavior moves further into the use of automated methods [40], TIBA could also be modified to include behaviors that are generated e. g. from automated posture classification [41] in its workflow. Doing so would again improve the ability of the researcher to detect patterns in the data which would otherwise be impossible without such a visualization tool. A comparison of automatically and manually annotated behavior could also lead to new insight regarding data collection and behavior. If standardized methods of gathering behavioral data are introduced, the comparison among species becomes feasible. Exploration of the similarities and differences in the behavioral sequences within and across species (or individuals) are already achievable with TIBA, but an assessment of the behaviors and behavioral transitions that are most decisive for the differences, respective to the applied distance measure, would be beneficial. Also, the explicit addition of phylogenetic approaches would vastly increase our power to compare behavioral sequences across species boundaries, generating great insight into the evolution of animal behavior.

Another important direction would be the evaluation of the tool itself. Through both controlled laboratory studies as well as case studies in real scenarios together with practitioners, we could assess and improve both the user experience and the practical usefulness of TIBA.

Future directions could involve the development of TIBA towards an immersive analytics [42] application which would offer researchers more intuitive and engaging ways to explore and interact with behavioral data, potentially through virtual or augmented reality environments. Additionally, developing collaborative approaches like ContextUWall [43], a system designed for collaborative analysis and visualization of data on multiple displays, could facilitate team-based research approaches, enabling collaborative exploration of behavioral data sets. These advancements could enhance TIBA’s utility for collaborative research projects, educational purposes, and interactive data exploration.

## Supporting information

S1 Dataset Sample data for *Neolamprologus multifasciatus*. (XLSX)

S2 Dataset Sample data for *Lamprologus ocellatus*. (XLSX)

S3 Dataset Sample data for *Telmatochromis temporalis*. (XLSX)

S4 Dataset Comparison data 1 for *Neolamprologus multifasciatus*. (XLSX)

S5 Dataset Comparison data 2 for *Neolamprologus multifasciatus*. (XLSX)

S6 Dataset Comparison data 3 for *Neolamprologus multifasciatus*. (XLSX)

S7 Dataset Comparison data for *Neolamprologus multifasciatus*. (XLSX)

S8 Dataset Comparison data 5 for *Neolamprologus multifasciatus*. (XLSX)

S9 Dataset Comparison data 6 for *Neolamprologus multifasciatus*. (XLSX)

S10 Dataset Comparison data 7 for *Neolamprologus multifasciatus*. (XLSX)

S11 Dataset Comparison data 1 for *Lamprologus ocellatus*. (XLSX)

S12 Dataset Comparison data 2 for *Lamprologus ocellatus*. (XLSX)

S13 Dataset Comparison data 3 for *Lamprologus ocellatus*. (XLSX)

S14 Dataset Comparison data 4 for *Lamprologus ocellatus*. (XLSX)

S15 Dataset Comparison data 5 for *Lamprologus ocellatus*. (XLSX)

S16 Dataset Comparison data 6 for *Lamprologus ocellatus*. (XLSX)

S17 Dataset Comparison data 7 for *Lamprologus ocellatus*. (XLSX)
